# Performance of Noise Reduction and Skid Resistance of Durable Granular Ultra-Thin Layer Asphalt Pavement

**DOI:** 10.3390/ma13194260

**Published:** 2020-09-24

**Authors:** Wei Li, Sen Han, Qibo Huang

**Affiliations:** 1 Highway and Airport Pavement Research Center, School of Highway, Chang’an University, Xi’an 710064, China; lhdww_forever@chd.edu.cn; 2 School of Transportation, Southeast University, Nanjing 210096, China; huangqibo@seu.edu.cn

**Keywords:** asphalt mixture, ultra-thin layer, durability, noise reduction, skid resistance

## Abstract

In this paper, the ultra-thin layer (UTL) is defined as the dense framework structure mixture made of asphalt binder, fine aggregate with nominal maximum aggregate size (NMAS) not greater than 13.2 mm and possible additives (mineral or organic), thickness of 2–4 cm. The study aims to investigate comprehensive performance of UTL asphalt mixture. The method of impact freeze thaw split test and the index of impact freeze–thawing damage degree (IFTDD) are proposed to reflect the durability. The indoor tire-rolling-down test system and accelerated abrasion machine are used to simulate the tire-pavement interaction and test road noise and skid resistance, respectively. Though evaluating the influencing factors (pavement thickness, gradation, asphalt binder type, and the content of KS additive) on durability, the optimum parameters with excellent durability are recommended. Combined with the test of noise and skid resistance, the factors affecting the surface function are analyzed. Moreover, the prediction mathematical model of skid resistance and the long-term safety benefit value E_eff_ are put forward. Results indicate that pavement thickness is the most significant factor effecting on durability, and gradation is the most significant factor affecting noise. Compared with KS additive, gradation has a greater influence on skid resistance index of Texture Depth (TD), whereas, KS additive is the most significant factor affecting British Pendulum Number (BPN). Furthermore, with the addition of asphalt rubber (AR), IFTDD and noise are reduced by 29.17% and 1.6 dB, and BPN and TD increase by 0.7 and 0.03 mm, remarkably. Compared with different asphalt types, the noise of UTL asphalt rubber mixture with 13.2 mm NMAS (UTL13 AR) is the lowest. Additionally, when KS content increases by 0.6%, the noise increases by 3 dB. Furthermore, on the basis of the calculation results of E_eff_, UTL13 AR mixture with 0.5% KS has the best long-term benefit of pavement safety and is recommended for field project.

## 1. Introduction

Recently, in addition to the traditional requirements of rutting resistance, cracking resistance and moisture susceptibility, the durability, safety and environmental protection of pavement are being paid more and more attention. Tire/road noise is one of the major environmental problems of modern society [1]. How to construct asphalt pavement with excellent using function under limited financial and material resources is also one of the main problems faced by highway development. Because of the prominent economic and social benefits and good performance, the thin layer surfacing asphalt pavement has been popularized rapidly.

The influence factors of pavement durability are very complex. Traffic load and environmental conditions are the two most important factors. At present, there are many researches on the durability of asphalt mixture at home and abroad. Most of the existing researches focused on the optimization of gradation and the indexes improvement of conventional road performance. Tan et al. evaluated the low-temperature performance by froze broken temperature and froze broken strength [2]. Cui et al. evaluated the durability of asphalt mixture though analyzing the effect of water on the adhesion of three aggregates by the index of adhesive fracture energy [3]. Yang et al. concluded that interlayer shearing stress of ultra-thin wearing course was affected by contact conditions and temperature [4]. Pavements fail because asphalt binders harden due to oxidative and thermo-reversible processes. Angius et al. provided a comparison between various measures of durability of 24 asphalt binders, and found the limiting phase angle temperature could accurately predict thermo-reversible aging tendencies [5]. Berkowitz et al. evaluated the effects of oxidative and thermo-reversible aging on the intermediate limiting phase angle temperatures and concluded that the effects of irreversible/chemical and thermo-reversible aging on rheological properties were comparable for fatigue cracking performance grading [6]. Due to the abundant connected pores and high impact failure energy of unit thickness under traffic load, the durability of Ultra-thin layer (UTL) pavement is an important aspect to address when designing the type of mixture. It has a great impact on economic and social benefits. Therefore, how to evaluate the long-term performance of asphalt pavement under the conditions of repeated load, water damage and ambient temperature needs to be solved.

Moreover, the tire/road noise is a significant contributor to the environmental pollution. It is not only related to the surface texture [7], but also to the basic material properties. Therefore, for studying noise reduction of UTL pavement further, it is critical to determine the links between the road material properties and the noise levels [8]. Previous studies have shown that at the same vehicle speed, the tire-pavement noise of rubber-modified asphalt Open Graded Friction Course (OGFC) mix was lower than that of Stone Mastic Asphalt (SMA) mix with nominal maximum aggregate size (NMAS) of 12.5 mm [9]. Jing et al. proposed that when the thickness of a porous asphalt (PA) concrete surface course was 8 cm, the noise reduction was the best [10]. The tire-pavement noise based on Mean Profile Depth (MPD) has been studied by many scholars. The results indicated that aggregate gradation evidently affected the MPD and tire/road noise [11]. Additionally, many test methods including close proximity method (CPX) and on-board sound intensity (OBSI) method have been proposed and applied for measuring Tire-Pavement noise at the Source [12,13,14]. Most of the tire/road noise tests are conducted on road sections. In such a situation, noise-influencing parameters cannot be well considered [15].

Furthermore, due to inadequate surface friction, the uncontrolled skidding causes 20% to 35% of all weather accidents, and wet accident rate is inversely proportional to skid resistance for all categories [16]. Alternatively, 70% of the wet weather crashes are preventable with improved pavement surfacing texture or friction, which is generally controlled by the choice of asphalt mixes including aggregate gradation and size [17]. Liu et al. showed that the macro-texture affected the attenuation magnitude of anti-skid performance at different driving speeds in wet weather [18]. Guan et al. designed and evaluated the long-term anti-skid performance of SMA16 thin-layer asphalt pavement [19]. Liu and sun considered that coating-type color anti-skid pavement has good application prospect in traffic safety [20]. Cong and Wang investigated fine aggregate angularity and found that it had a significant impact on the macro-texture of skid-resistance, but only a marginal influence on the micro-texture [21].

Despite the research found on the durability, noise reduction and skid resistance, little previous study has been reported concerning the road performance comparison on the UTL pavement with different factors, such as pavement thickness, gradation type, asphalt binder type, additive content, etc. Therefore, this paper aimed to explore the surface function of UTL mixture. The aggregate gradation had been achieved in the prophase study of our research group [22].

The main study contents were as follows: (1) In accordance with the complex impact and shear state of vehicle load on UTL pavement, the corresponding evaluation method of durability was proposed; the different factors affecting durability were studied to provide the design basis for durable asphalt functional layer. (2) Though the methods developed in our laboratory, noise and skid resistance of UTL were measured, the influence factors on the surface function were analyzed. In this case, the noise reduction and skid resistance durability were evaluated. The optimum composition of UTL pavement that could effectively improve the service life, reduce tire/road noise and guarantee the traffic safety was obtained.

The objective of the study is to research new materials that meet the function requirements of thin surfacing asphalt pavement and have sufficient scientific and economic basis in order to provide technical guarantee and scientific basis for the promotion and construction of thin surfacing asphalt pavement.

## 2. Materials and Test Methods

### 2.1. Raw Materials

Aggregate: Coarse aggregate was diabase rolled crushed stone, fine aggregate was diabase machine-made sand, and mineral powder was made of limestone. Their properties met the requirements of Chinese specification JTG E20-2011 [23]. The main technical indexes of coarse aggregate are shown in Table 1. The apparent relative density of fine aggregate and mineral power was 2.729 and 2.714, respectively.

Asphalt binder: SK-90 was used as virgin asphalt in this research. Asphalt rubber (AR) and SBS modified asphalt were selected as two modified asphalt. The basic physical properties of the three asphalts were measured according to Chinese specification JTG E20-2011 [23]. The main technical indexes are shown in Table 2.

Additive: KS is a kind of asphalt mixture additive synthesized by chemical modification of a variety of polymer and nanometer waste residues. It has gray black flat solid particles and can be preserved at normal atmospheric temperature. The particle size, relative density and melting point were ≤6 mm, 0.92–0.97 and 140–160 °C, respectively. In the study, the KS contents of 0%, 0.3%, 0.4%, 0.5%, and 0.6% by the weight of asphalt mixture were selected.

### 2.2. Aggregate Gradation

**Figure 1:**
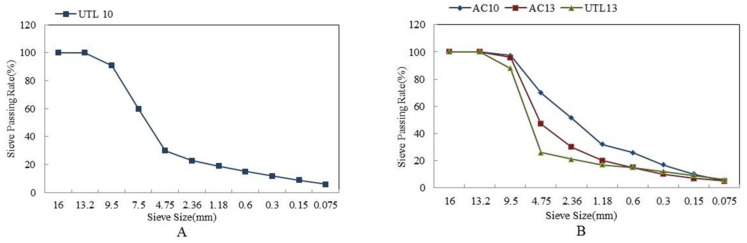
The gradation curves of different asphalt mixtures: (**A**) UTL10; (**B**) UTL13, AC13 and AC10.

Determination of optimum gradation of UTL asphalt mixture: The dense framework-gradation with 13.2 mm NMAS (UTL13) and 9.5 mm NMAS (UTL10) were selected. Combined with stone asphalt concrete (SAC) aggregate gradation design method [24], the proportions of coarse aggregate of UTL13 and UTL10 were recommended in the previous research, which put forward to add a 7.5-mm sieve between the 4.75 mm and 9.5 mm sieves to ensure the aggregate gradation stability of UTL10 [22]; the optimum gradations were determined for UTL13 and UTL10 by Marshall test volume indexes. Meanwhile, two kinds of suspended dense-graded asphalt concretes with 13.2 mm NMAS (AC13) and 9.5 mm NMAS (AC10) were selected for comparative study. The gradation of UTL10 is shown in Figure 1A; UTL13, AC13 and AC10 are shown in Figure 1B.

### 2.3. Test Methods

#### 2.3.1. Durability Test

Durability evaluation index: Cracking is generally related to material fatigue, possibly caused by repeated traffic loading. Moreover, thermal effects might provide the initiating and propagation mechanism for the evolution of many kinds of crack pattern [25]. Therefore, in order to simulate the effect of vehicle load, water damage and ambient temperature on UTL pavement, the indoor impact freeze thaw split test method was invented by our research group [26]. In this method, the experiment was designed with a control trial. The asphalt mixture specimens in the control group were directly subjected to splitting test. For the experimental group, the cyclic impact test, water damage test and temperature change test were carried out first to simulate the actual loss of pavement, and then the splitting test was conducted on the mixture specimens. The splitting strength of the control group and the experimental group was obtained respectively. Ultimately, the durability of thin-layer asphalt mixture was evaluated by the impact freeze-thawing damage degree (IFTDD) calculated according to Formula 1. The smaller value of IFTDD means the stronger ability to resist the coupling effects of load impact, water damage and ambient temperature.

(1) $$D=1-EE_{0}^{-1}$$

where: *D* is the IFTDD; *E* is the impact freeze-thaw splitting strength of experimental group (IFTSS); and *E*_0_ is the splitting strength of control group (SS) of asphalt mixture.

Specimen preparation: The standard Marshall specimens with diameter of 101.6 mm were prepared after two-side compaction of 75 times. The specimens were cut to three thicknesses (2 cm, 3 cm and 4 cm, respectively) with a diameter of 101.6 mm.

Selection of standard impact times: Marshall specimens with 3 cm thickness were tested at 25 °C under different impact times to ensure that the asphalt mixture reached the critical failure state after certain impact times and observed the cracking phenomenon of specimen surface.

Impact freeze thaw split test: The freeze/thaw cycling test referred to Chinese specification JTG E20-2011 [23]. Firstly, the specimens in the experimental group were frozen in a refrigerator at −12 °C ~ −8 °C for 23.5–24.5 h. Secondly, the specimens were fixed on the steel base and put into the heat preservation box keeping the low temperature of −12 °C to −8 °C. In the heat preservation box, the specimens were impacted by the counterweight hammer with a counterweight of 10 kg for 30 times. Then at room temperature, the specimens were put into a hollow container, soaked with water, and the container was closed for vacuumization. The specimens were kept in a vacuum of 97.3 kPa for 15–25 min and returned to normal pressure for 20–40 min. Next, the water saturated specimens were taken out, wrapped in plastic bags, and frozen in a low-temperature refrigerator at −18 °C for 15–17 h, put into a water bath with water temperature of 60° C for 22–26 h, and then into a normal temperature water tank for 2 h. Finally, the splitting test was carried out on the specimens.

#### 2.3.2. Tire/Road Noise Test Method

**Figure 2:**
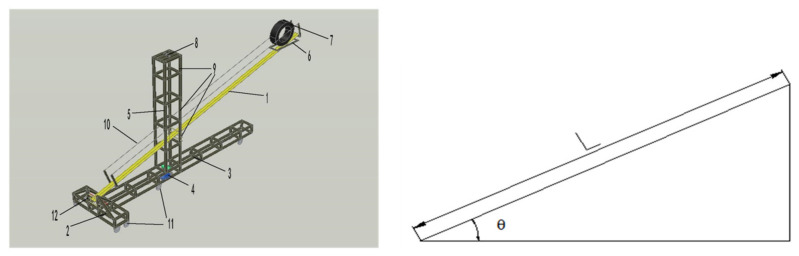
3D model of indoor tire-rolling-down test system and calculation diagram of tire falling speed.

The indoor tire-rolling-down test system invented by our research group was used to simulate the tire/road noise in the laboratory [15,27]. In the test process, the initial speed of test tire was zero, and the rolling track had a θ° angle with the horizontal plane. The tire was released from the holder and allowed to roll down freely from the top of the track to the bottom, and drop onto the test specimen. The horizontal speed of the tire hitting the test specimen simulated a forward speed on the car. The vertical velocity simulated the vehicle impact vibrations on the road surface. The sound pressure measurement devices used in this study included microphones, a sound digital signal acquisition instrument, data acquisition software, and an acoustic calibrator. In this research, the track length was 6 m, and horizontal inclination θ was 15°. The horizontal speed of the tire falling to the test specimen was 19.2 km/h, and the vertical speed was 5.1 km/h. The 3D model of indoor tire-rolling-down test system [15,27] and simplified model of tire falling process are shown in Figure 2.

Specimen preparation: according to Chinese specification JTG E20-2011 [23], the rut boards were prepared and used to analyze the influence of different factors on tire/road noise. The parameter combinations of rut board specimens are shown in Table 3.

#### 2.3.3. Skid Resistance Test Method

The accelerated abrasion machine invented by our research group [28] was used to simulate the effect of vehicles on pavement and test the skid resistance affected by different factors (asphalt binder types, gradation types and KS content). The BPN and TD were used as evaluation indexes. Test processing: Firstly, prepared asphalt mixture specimens had a size of 300 mm × 300 mm × 50 mm and measured initial BPN and TD according to the Chinese standards [23,29]. Secondly, the specimen was placed in the accelerated abrasion test bench, and the specimen was fixed after adjusting the position. Then the power supply was started and the test piece was loaded under 0.7 MPa of the tire pressure; the round-trip speed of the tire was 48 times/min to test BPN and TD every 1 h and obtain the skid resistance data in the wearing process lastly.

### 2.4. Significance Analysis of Each Factor

The Analysis of Variance (ANOVA) method is known as F-test. The basic idea is to determine whether the independent variables have significant influence on the dependent variables by testing whether the mean value of each population is equal. In ANOVA, if only one influencing factor is involved in a research, it is called one-way ANOVA. In this paper, SPSS statistical software was used to analyze the significance of different factors on durability, noise and skid resistance of UTL pavement. In the test process, the control variable method was used. Therefore, for the analysis of the impact of each factor on different road performances, the cross effect of different factors was not considered, and the one-way ANOVA was used. The F-value was used to evaluate the differences between groups. Each F-value corresponds to a value of *p*. The larger the F-value, the smaller the p-value, the greater the difference in characteristics between groups.

## 3. Experimental Results and Discussions

### 3.1. Durability

#### 3.1.1. Determination of Impact Times

**Figure 3:**
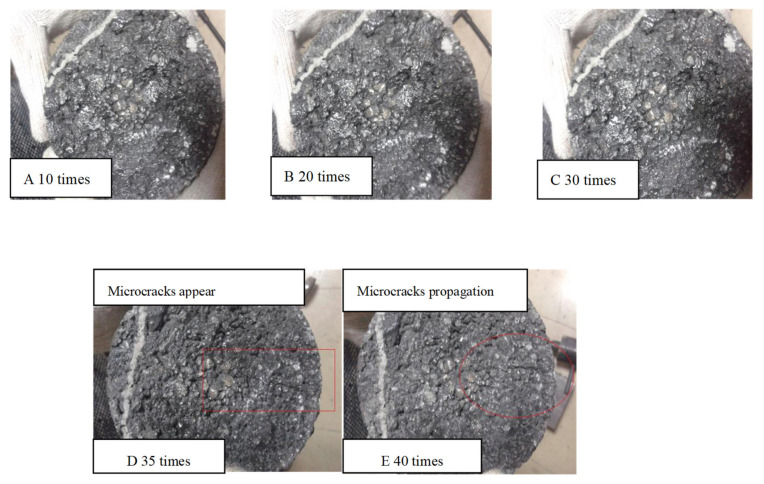
Cracking phenomenon of UTL under different impact times: (**A**) Impact 10 times; (**B**) Impact 20 times; (**C**) Impact 30 times; (**D**) Impact 35 times; (**E**) Impact 40 times.

The impact test results are shown in Figure 3. It can be seen from Figure 3 that there were no obvious cracks on the mixture surface with impacts 0–30 times. However, after 35 times, micro cracks appeared clearly on the surface of specimens. When the impact lasted for 40 times, the micro cracks expanded and the specimens were destroyed. Moreover, according to the phenomenon that micro cracks appeared on the top of the test piece but the structure at the bottom was complete, it could be inferred that top-down crack was also one of the failure forms of UTL asphalt pavement with reasonable structural strength. This was consistent with previous findings that the ‘primary mechanism of top-down cracking’ in asphalt pavement ‘accumulated damage associated with repeated traffic loading’ [30]. Therefore, 30 times was determined as the standard impact times for the following study.

#### 3.1.2. Effect of Pavement Thickness on Durability

**Figure 4:**
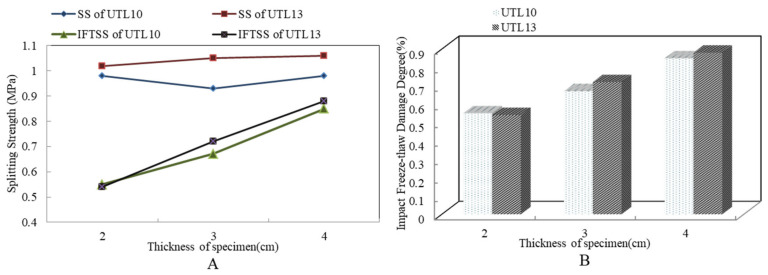
Results of impact freeze thaw split test with different mixture thicknesses: (**A**) SS and IFTSS; (**B**) IFTDD.

Figure 4 shows the impact freeze thaw split test results of asphalt mixture with different mixture thicknesses. From Figure 4A, SS hardly changed when the thickness increased, while the IFTSS and the IFTDD increased obviously. When thickness increased from 2 cm to 4 cm, IFTDD was reduced by 30.08%. The main reasons were: (1) when the thickness decreased, the connectivity porosity of asphalt mixture increased. It provided more interior space for water absorption and frost heave. Under the effect of freeze/thaw cycling, the probability of micro damage in asphalt mixture was vastly increased. With the occurrence of large-scale damage, the internal structure of asphalt mixture was weakened so that the structural strength decreased. (2) With the increase of specimen thickness from 2 cm to 4 cm, the IFTSS increased by 0.16 MPa at most. Under the equal impact failure energy, the impact energy of unit thickness specimen declined with specimen thickness increasing, and the relative stability of the internal structure could be guaranteed under the lower impact failure, which showed that the IFTSS of UTL increased with the increase of specimen thickness. In addition, according to Figure 4B, there was a good correlation between IFTDD and mixture thickness. Through comparison, it was found that the IFTDD with different thicknesses was about 15–45%, the maximum IFTDD was close to 50%. Therefore, the test had a good distinguishing effect to characterize the comprehensive effect of load and freeze/thaw environment on durability of UTL asphalt mixture.

#### 3.1.3. Effect of Aggregate Gradation Type on Durability

**Figure 5:**
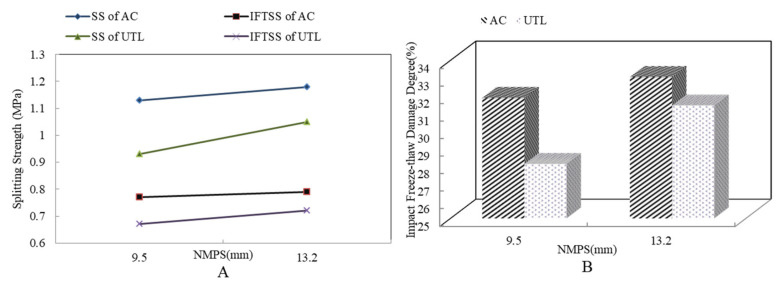
Results of impact freeze-thaw spilt test with different NMAS: (**A**) SS and IFTSS; (**B**) IFTDD.

Figure 5 shows the results of impact freeze thaw split test of asphalt mixture with different NMAS. It is illustrated from Figure 5A with the decrease of NMAS, the SS and IFTSS tended to decline. The IFTSS of AC10 and UTL10 was 0.02 MPa and 0.05 MPa lower than that of AC13 and UTL13, respectively. It was because when the air voids of the mixture were similar, the internal friction and the structural strength were improved with NMAS increasing. The IFTSS of four mixtures was: AC13 > AC10 > UTL13 > UTL10. It did not mean that the strength of the suspended dense-graded asphalt mixture was higher than that of the dense framework graded asphalt mixture, only because the splitting test had a difference between the loading effect of the splitting test and the actual stress state of the pavement. 

From Figure 5B, The IFTDD of four asphalt mixtures was: UTL10 < UTL13 < AC10 < AC13. The IFTDD with the same NMAS and different gradation types was obviously different. Compared with AC, UTL had a lower damage degree because the internal structure had stronger skeleton supporting function. When the mixture was impacted by external force, the coarse aggregate could resist the damage and deformation better. It ensured the structure integrity of UTL pavement. Conversely, due to the lower structural strength, more micro cracks and greater structural damage occurred in AC under the action of impact. Once the skeleton structure of the dense framework structure asphalt mixture was damaged, a large range of cracks would appear, under the extreme water and temperature actions, it showed a greater degree of damage. Generally, the aggregate gradation determines the density of the mixture and the embedded force between the aggregate particles [31]. The smaller the NMAS was, the less possibility of freeze-thawing damage to mixture structure was. With the decrease of NMAS, the pore diameter was smaller, so that the more closed void structure were formed and the smaller IFTDD was generated.

#### 3.1.4. Effect of Asphalt Binder on Durability

**Figure 6:**
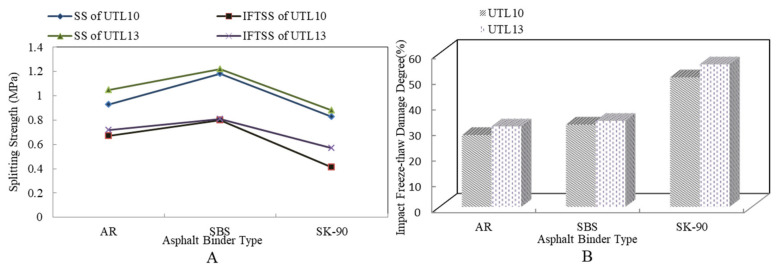
Results of impact freeze-thawing spilt test with different asphalt binders: (**A**) SS and IFTSS; (**B**) IFTDD.

Figure 6 shows the results of the impact freeze thaw split test of asphalt mixture with different asphalt binders. It is demonstrated that from Figure 6A under the same test conditions, the splitting strength of SBS modified asphalt mixture was always greater than that of AR mixture. The SS and IFTSS of different asphalt mixtures was: SBS modified asphalt mixture > AR mixture > virgin asphalt mixture. It showed that SBS asphalt mixture was more sensitive to water and low temperature than AR mixture [32]. From Figure 6B, the IFTDD of different asphalt mixtures was: AR mixture < SBS modified asphalt mixture < virgin asphalt mixture. This result proved that AR mixture had the strongest impact freeze-thawing resistance. Compared to virgin asphalt, IFTDD of AR mixture reduced 29.17%.

Asphalt binder is an important component of pavement materials. The high durability of asphalt binder will prevent premature failures in terms of fatigue, low temperature cracking and moisture damage [5]. Despite some scholars having claimed that a decrease in strength and resistance to penetration was observed at high temperature, SBS increased the low-temperature flexibility and elasticity of asphalt [33,34]. In the freeze thaw split test, the indirect tensile force mainly acted between the aggregate particles in specimens. Due to the good ductility at low-temperature, SBS modified asphalt mixture had strong tensile strength and was not easy to be destroyed. As a result, the splitting strength measured of SBS mixture was larger than that of other asphalt mixtures. After a long period of freeze/thaw cycling, compared with AR mixture, SBS-modified asphalt was more sensitive to water and low-temperature. It resulted in a faster decline in splitting strength [32]. Therefore, AR mixture had the least structural damage after impact freeze thaw split test and AR was recommended as asphalt binder for durable UTL pavement.

#### 3.1.5. Effect of KS Content on Durability

**Figure 7:**
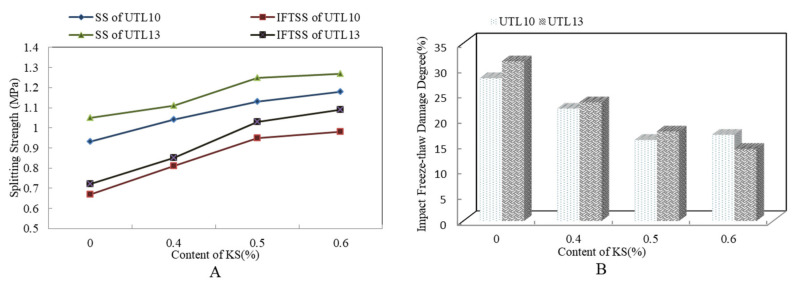
Results of impact freeze-thawing spilt test with different KS contents: (**A**) SSW and IFTSS; (**B**) IFTDD.

Figure 7 shows the results of impact freeze thaw split test of asphalt mixture with different KS content. It is found from Figure 7A that both SS and IFTSS of UTL13 AR mixture with 0.6% KS were the highest. When KS content increased from 0% to 0.6%, the SS increased 0.22 MPa. Furthermore, from Figure 7B, with the increase of KS content, the IFTDD declined obviously. When the content of KS exceeded 0.5%, the downward trend of IFTDD tended to moderate, and the improvement effect of KS on durability was weakened. The main reason was after KS was melted on the aggregate surface, and the effect of tackifying, reinforcing and inlaying between aggregates was produced, the deformation and damage of mixture under the impact load was restricted. Ultimately, the micro-cracks caused by impact were reduced so that the mixture still had strong water resistance in the freeze/thaw cycling test. According to the analysis, the content of KS was recommended to be 0.5% by the weight of asphalt mixture.

### 3.2. Noise Reduction of UTL

#### 3.2.1. Effect of Aggregate Gradation on Tire/Road Noise

**Figure 8:**
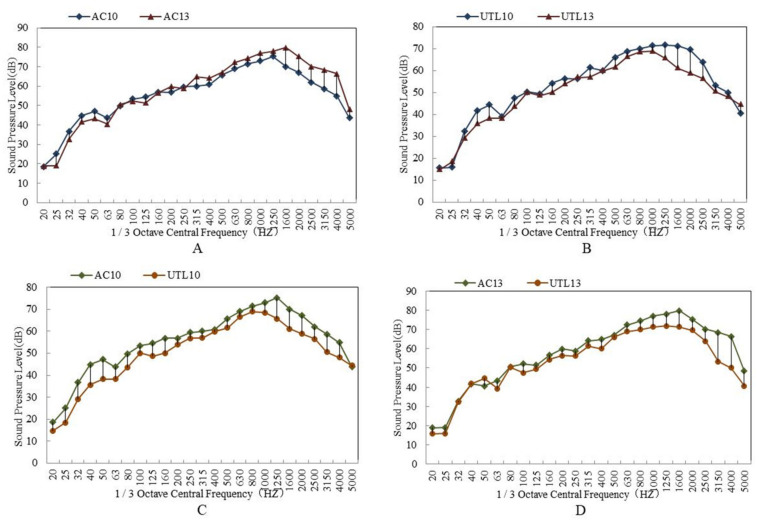
1/3 octave band spectrum of asphalt mixture with different aggregate gradations: (**A**) Suspended dense gradation AC; (**B**) Dense framework gradation UTL; (**C**) 9.5 mm NMAS; (D) 13.2 mm NMAS.

The tire/road noise test results of asphalt mixture with different gradation are shown in Table 4. The 1/3 octave band spectrum is shown in Figure 8. From Table 4, it is concluded that with the increase of NMAS, the tire-pavement noise increased. When NMAS increased from 9.5 mm to 13.2 mm, noise increased by 2.5 dB. Compared with AC10 and UTL10, the sound pressure levels of AC13 and UTL13 were higher by 4.4 dB and 2.5 dB, respectively. Moreover, the sound pressure levels of UTL10 and UTL13 were lower by 1.3 dB and 3.2 dB than that of AC10 and AC13.

As can be seen from Figure 8A,B, the 1/3 octave band spectrum of tire/road noise was a continuous spectrum; the range of frequency distribution was 200–5000 Hz. It was in the frequency area sensitive to human hearing [35]. With the increase of NMAS, the sound pressure level increased. Particularly, in the frequency range of 1100–1600 Hz, the difference of sound pressure between two classes mixture with different NMAS increased sharply. Generally speaking, tire/road pump noise was generated in the frequency range of 1100–1600 Hz, whereas tire/road vibration noise was produced in the frequency range of 600–1000 Hz. Therefore, reducing NMAS could effectively reduce the noise [36]. There are two reasons: On one side, with the decrease of NMAS, the micro-connected pores formed in asphalt mixture increased. Due to the fact that micro connected pores had a strong ability to absorb sound waves [37], the pump noise was reduced. On the other side, when the road surface area was the same, with NMAS having decreased, the wedge ends on the road surface were more, and the noise was reflected at the wedge ends many times. As a result, the acoustic energy was consumed quickly.

From Figure 8C,D, it is found that the maximum difference of 1/3 octave band spectrum between AC and UTL pavement mainly lied in the frequency range (1100 Hz–1600 Hz) of pump noise. This is because there were less connected pores in the suspended dense asphalt mixture AC, while the dense framework structure asphalt mixture UTL had more abundant surface texture and connected pores to release the compressed air between tire and road surface in time, which effectively reduced the suction pump noise.

#### 3.2.2. Effect of Asphalt Binder Type on Tire/Road Noise

**Figure 9:**
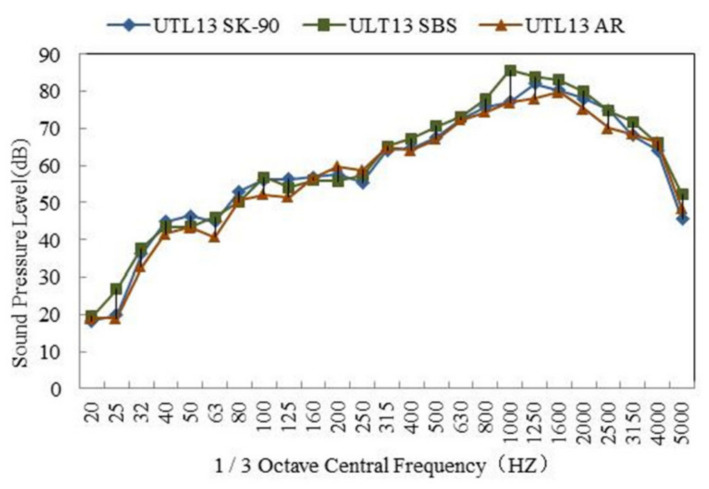
1/3 octave band spectrum of UTL with different kinds of asphalt binder.

The tire/road noise test results of different asphalt mixtures are shown in Table 5. The 1/3 octave band spectrum is shown in Figure 9. According to Table 5, the noise of different asphalt mixture was: AR mixture < virgin asphalt mixture < SBS modified asphalt mixture. The noise of AR mixture was the lowest, the sound pressure level was 80.1 dB. It was lower by 1.6 dB than that of virgin asphalt mixture. Furthermore, from Figure 9, it is indicated that the spectrum differences of three kinds of asphalt mixture were mainly concentrated in the frequency range of 800–1200 Hz. Compared with SBS asphalt mixture, the spectrum of AR mixture corresponding to the peak frequency transited from 1200 Hz to 800 Hz, and the peak value of sound pressure level increased obviously [37]. Because the peak frequency was in the range of vibration noise, the asphalt type mainly affected the dissipation of vibration noise. Among three kinds of asphalt pavement, AR pavement had the most evident effect on reducing vibration noise. Rubber powder had large damping, it could increase the damping coefficient of asphalt and enhance the elimination effect of vibration noise. As a result, acoustic energy was dissipated quickly and the tire/road noise was reduced. This was also the main reason why rubber asphalt pavement was called ‘low-noise pavement’.

#### 3.2.3. Effect of KS Content on Tire/Road Noise

**Figure 10:**
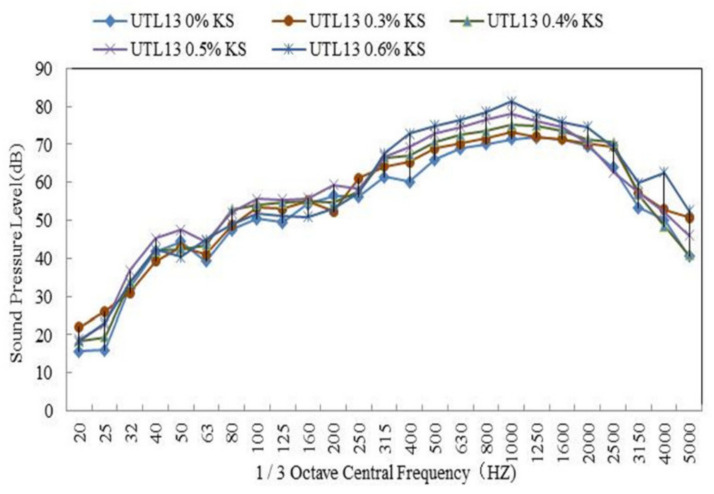
1/3 octave band spectrum of UTL with different KS contents.

The noise test results of asphalt mixture with different KS content are shown in Table 6. The 1/3 octave band spectrum analysis is shown in Figure 10. From Table 6, the noise of UTL13 pavement with different KS contents was: UTL13 0.6%KS < UTL13 0.5%KS < UTL13 0.4%KS < UTL13 0.3%KS < UTL13 0%KS. It is illustrated from Figure 10 that the noise of AR mixture without KS was the lowest. With the increase of the KS content, the tire-pavement noise increased. When the content of KS was 0.6%, the sound pressure level was higher by 3.0 dB than that of the asphalt pavement without KS. Additionally, it can be deduced that the KS additive mainly caused noise in the frequency range of 800–1200 Hz. With KS content having increased, the sound pressure level in the low frequency range (800–1200 Hz) increased, and the peak value relatively increased. The addition of KS content weakened the effect of AR mixture on vibration noise reduction. This was mainly because the KS additive increased the stiffness and reduced the damping of asphalt mixture by tackifying, reinforcement and inlaying among aggregate, which led to the increase of tire/pavement vibration noise.

#### 3.2.4. Effect of Pavement Thickness on Tire/Road Noise

**Figure 11:**
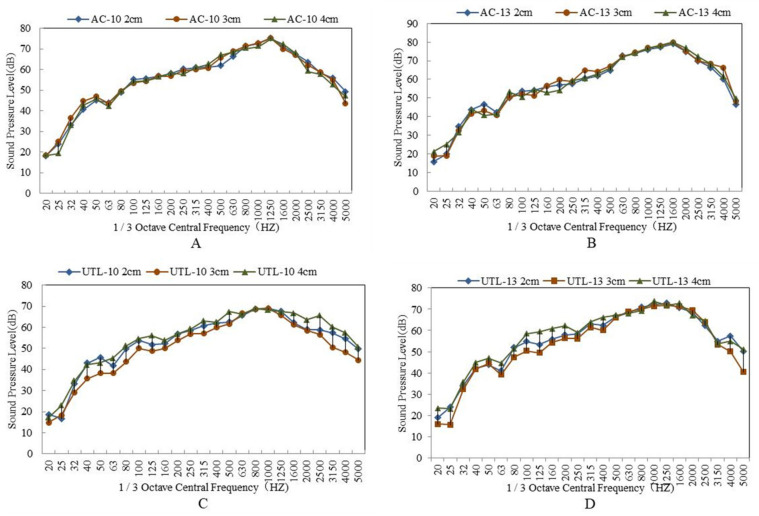
1/3 octave band spectrum of asphalt mixture with different pavement thickness: (**A**) AC-10; (**B**) AC-13; (**C**) UTL-10; (**D**) UTL-13.

The noise test results of asphalt mixture with different mixture thicknesses are shown in Table 7. The 1/3 octave band spectrum is shown in Figure 11. It is illustrated from Table 7 that there was no evident difference in tire/road noise among different pavement thicknesses. The noise of two kinds of pavement was: AC > UTL. Compared with AC pavement, the noise of UTL was lower by 1–3 dB. When thickness increased from 2 cm to 4 cm, noise was only reduced by 0.3 dB. On the basis of Figure 11, the change of pavement thickness did not affect the peak noise and peak frequency, and there was no obvious change of sound pressure level. Therefore, it was shown that the pavement thickness was not the main factor affecting the noise of UTL pavement.

### 3.3. Skid Resistance

#### 3.3.1. Effect of Aggregate Gradation on Skid Resistance

**Figure 12:**
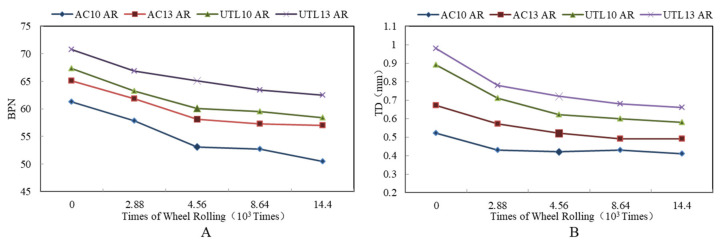
Attenuation rules of skid resistance indexes with different gradations: (**A**) BPN; (**B**) TD.

Microtexture is primarily a function of aggregate surface characteristics, whereas macrotexture primarily depends on aggregate gradation and method of construction [38]. It is illustrated from Figure 12A that in the initial stage of wheel loading, BPN was relatively larger. With the increase of the loading times, BPN decreased gradually, and the attenuation amplitude also tended to moderate. After loading times of 8.64 × 10^3^–14.4 × 10^3^, the BPN was basically stable. Moreover, the change trends of BPN of asphalt mixture with different gradations were essentially the same. The order of BPN attenuation characteristics of asphalt mixture was: UTL > AC.

As can be seen from Figure 12B, with loading times increasing from 0 to 2.88 × 10^3^, the TD of different gradations declined remarkably. After loading times of 4.56 × 10^3^, TD tended to be steady. The change trend of skid resistance showed under the action of tire, asphalt mixture was compacted first, which led to the change of TD. The loading times corresponding to this process were 0–4.56 × 10^3^. When loading times continued to increase, the downward trend of TD tended to ease up. Meanwhile, the final value of TD also reflected the difference of structural stability between different asphalt mixtures. Under the compression of wheel loading, the asphalt mortar floated on the mixture surface, resulting in the decrease of TD. This was consistent with the previous study [39]. Compared to AC, although the asphalt mortar of UTL mixture floated more, the final attenuation value of TD was greater due to the larger content of coarse aggregate. Moreover, UTL belonged to dense framework structure, so the initial value of TD was larger, the attenuation was slower in the process of wheel loading, and the final value of attenuation was also larger. In addition, the asphalt mixture with 13.2 mm NMAS had better skid resistance performance than that of 9.5 mm NMAS, and the TD attenuation rate was lower in the process of abrasion test. It led to a higher attenuation final value of TD. Therefore, increasing NMAS could effectively enhance the skid resistance of asphalt mixture.

#### 3.3.2. Effect of Asphalt Binder on Skid Resistance

**Figure 13:**
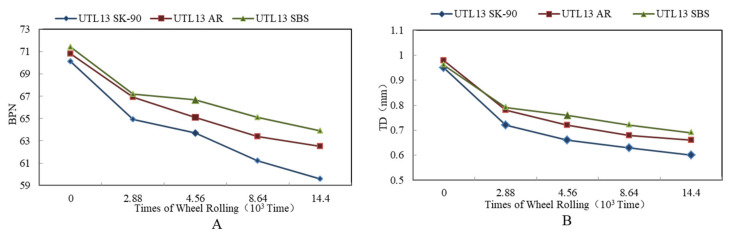
Attenuation rules of skid resistance indexes with different asphalt binder types: (**A**) BPN; (**B**) TD.

It is found from Figure 13 that BPN and TD of three mixtures was: SBS asphalt mixture > AR mixture > virgin asphalt mixture. Compared to virgin asphalt mixture, BPN and TD of AR mixture increased 0.7 and 0.03 mm respectively. From Figure 13A, in the initial stage of skid resistance attenuation, BPN of three types of asphalt mixture all dropped rapidly to a certain value. After loading times of 4.56 × 10^3^ times, with the increase of loading times, the attenuation rate of skid resistance slowed down. Similarly, as can be seen from Figure 13, TD of different asphalt mixtures decreased with the change of loading times. The change trend of TD declined rapidly in the initial stage and then tended to moderate. According to raw material properties of three kinds of asphalt, SBS modified asphalt had the best capacity to cement aggregate, and the deformation resistance was higher than that of AR. The cementation strength was: SBS > AR > SK-90 virgin asphalt.

#### 3.3.3. Effect of KS Content on Skid Resistance

**Figure 14:**
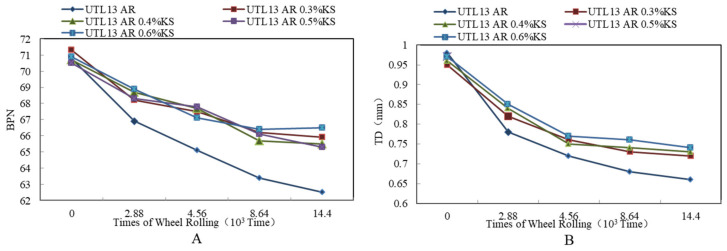
Attenuation rules of skid resistance indexes with different KS contents: (**A**) BPN; (**B**) TD.

It is illustrated from Figure 14A that there was the same attenuation law of BPN between the asphalt mixtures with KS and without KS. In the initial stage of wheel loading, BPN decreased significantly and then tended to be stable. Compared to asphalt mixture without KS, the fast attenuation phase of BPN of the asphalt mixture with KS was shorter, and the attenuation amplitude was relatively smaller and had no obvious monotonous relationship with the increase of KS content. According to Figure 14B, the change rule of TD was similar to BPN. With the increase of loading times, TD decreased significantly first and then tended to be stable.

#### 3.3.4. Long Term Model and Durability Benefit Evaluation of Skid Resistance

It can be seen from Section 3.3.1–Section 3.3.3 that the skid resistance of asphalt mixture followed the attenuation law. The initial value decreased rapidly and tended to be steady gradually. Therefore, the statistical principle was used to fit the mathematical model of the attenuation law of skid resistance durability. The skid resistance durability of mixture was evaluated theoretically by a mathematical model. Test materials and test conditions were the main factors affecting test results of skid resistance. In the mathematical model, the change of materials and test conditions were reflected by corresponding parameters. A large number of tests and engineering data showed that the skid resistance attenuation law of asphalt pavement obeyed ‘s’ function distribution [40]. The characteristic of ‘s’ function model is that the change speed is fast in the initial stage and tends to be gentle when it is close to the critical value. The change process of model function is consistent with the attenuation law of pavement skid resistance. Formula (2) is for function equation.

(2) $$PPI=a+\frac{b-a}{1+e^{\beta +\mathrm{kt}}}$$

where: *PPI* is skid resistance performance index; *a* is maximum value of skid resistance index; *b* is minimum value of skid resistance index; t is operation period; and β and k are regression parameters.

1. Durability model of BPN.

The BPN is generally less than 80. In the Chinese specification [41], when the BPN of Expressway and First grade highway are less than or equal to 37, measures should be taken to restore the skid resistance of pavement. Therefore, the model parameters of ‘a’ and ‘b’ were 80 and 37 in the study, respectively. According to Formula (2), the mathematical model of BPN was obtained as Formula (3).

(3) $$BPN=a+\frac{b-a}{1+e^{\beta +\mathrm{kt}}}$$

Transferred Formula (3) to Formula (4):

(4) $$\ln\frac{BPN-b}{a-BPN}=\beta +\mathrm{kt}$$

**Figure 15:**
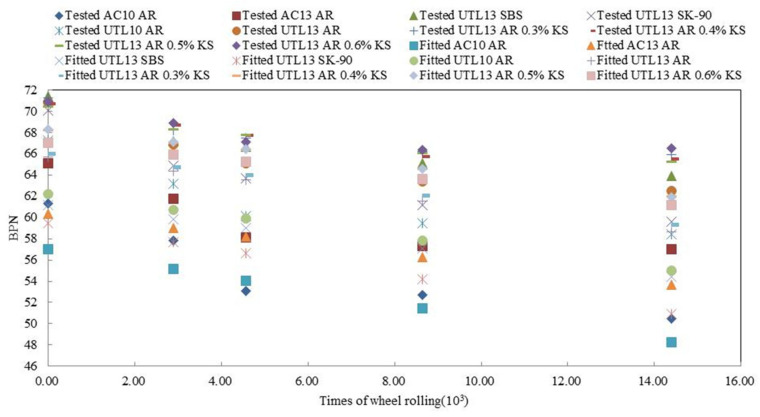
Comparison between the model calculation values and the tested values of different asphalt mixture.

Supposed Y*_BPN_* = ln ((*BPN* − *b*)/(*a* − *BPN*)) and gained Y_BPN_ = β + kt. According to the test results, regression analysis was carried out on the calculation results of Y_BPN_, and the model calculation parameters of BPN in different test schemes were obtained. The results are shown in Table 8. A comparison between the fitted value and the measured value of different asphalt mixtures is shown in Figure 15. As can be seen from Figure 15, for BPN of asphalt pavement, the calculated values of the mathematical model and the tested values had a high fitting degree. From the final stage and the overall forecast trend, the prediction model of ‘s’ function conformed to the actual situation. Therefore, ‘S’ function was a suitable prediction model for BPN attenuation.

2. Durability model of TD.

The initial TD of common asphalt pavement was not more than 1.5. Referencing to the Chinese standard JTG F40-2017 [42], for high-grade asphalt pavement, the TD should be more than or equal to 0.55 mm in completion acceptance. Therefore, model parameters of ‘a’ and ‘b’ were 1.5 and 0.55 respectively. The model calculation parameters of TD for different asphalt mixtures are shown in Table 9.

3. Evaluation of durability benefit of skid resistance

In order to further analyze the influence of asphalt mixture type and KS additive on safety of skid resistance, the ‘long-term benefit index’ of pavement durability of safety was used to evaluate the safety effect [43] of different asphalt mixtures. The calculation method of long-term safety benefit index of pavement is shown in Formula (5) and (6).

(5) $$E_{avg}=\frac{1}{T_{\mathrm{life}}}(y_{0}+y_{1}+y_{2}+\sim +y_{c-1}+y_{c})$$

(6) $$E_{eff}=\frac{E_{avg}-E_{INI}}{E_{INI}}$$

where *E_avg_* is the mean value of pavement performance index in service life of asphalt pavement; *T_life_* is service life; y_0_ is the initial performance index of new-built pavement; y_1_ is the performance index of pavement during different service periods; *y*_c_ is the performance index at the last period of pavement service life; *E_eff_* is the long-term benefit of pavement safety%; and *E_INI_* is the lower limit of pavement performance index according to specification. Through the prediction model, the long-term benefits of skid resistance were obtained. The calculation results are shown in Table 10.

From Table 10, it is illustrated that KS admixture could significantly improve the durability of skid resistance. For BPN, the maximum difference of loading times in whole service life between the asphalt mixtures with KS and without KS was 15 × 10^3^ times. Furthermore, for the skid resistance indexes of BPN and TD, due to the different attenuation mechanisms, the durability life prediction was different. Therefore, when the service life of asphalt pavement was comprehensively evaluated, the appropriate evaluation index should have been selected according to the use occasions of asphalt pavement. Additionally, the skid resistance durability of asphalt mixture with different gradations was different. The durability of the dense framework structure UTL was better than that of suspended dense structure AC. On this basis, considering that the dense framework gradation rubber-modified asphalt mixture with KS additive had excellent skid resistance durability, the UTL AR pavement with 13.2 mm NMAS and 0.5% KS was recommended for field project.

### 3.4. Significance Analysis of Each Factor

The ANOVA results of different influencing factors on durability, noise and skid resistance are shown in Table 11. From Table 11, for durability, the *p*-values corresponding to four factors were less than 0.001. It indicated that the four factors had very significant impact on durability of UTL pavement. Furthermore, when the freedom degrees are the same, with the F-value increasing, the *p*-value decreases. According to F-values, the order of the significance of different factors on durability was: pavement thickness > asphalt type > KS content > gradation. Therefore, compared with the other three factors, pavement thickness was the most important for the durability design of UTL pavement. However, for tire/road noise, the *p*-value corresponding to pavement thickness was 0.114, which was more than 0.05. It showed that the influence of pavement thickness on noise of UTL pavement was not significant. Moreover, according to the fact that *p*-values corresponding to asphalt type, gradation and KS content were all less than 0.001, it was found that these three factors had significant influence on tire/road noise. Combined with F-values, the importance of different factors on noise of UTL pavement was: gradation > KS content > asphalt type > pavement thickness, and the significance of KS content and asphalt type on noise was equivalent. Therefore, in the design of UTL pavement, the influence of aggregate gradation on noise reduction should be fully considered. For the skid resistance index of BPN, the *p*-values corresponding to different factors were all less than 0.001. Combined with the F-values, the order of importance of the three factors was: KS content > asphalt type > gradation. In addition, the significance of KS content was equivalent to that of asphalt type. However, for TD, the *p*-value corresponding to asphalt type was 0.147, which was more than 0.05. It indicated that the influence of asphalt type on structure depth was not significant. According to F-values, the order of the importance of different factors was: gradation > KS content > asphalt type. The most likely reason is that BPN represents the friction resistance of pavement in wet state, it is easily affected with the change of KS content, asphalt type and aggregate gradation, whereas TD as a macro index of pavement structure characterizes the depth of open pores on a certain area of road surface, and it is little affected by asphalt types. Compared to other factors, the effect of KS additive on the comprehensive performance of mixture was limited. Therefore, considering the economic benefits, the additive content should be suitable, not the higher the better. For pavement thickness and gradation, it was necessary to select more appropriate parameters to meet the engineering requirements and economic benefits on the premise of improving the pavement performance to the greatest extent.

## 4. Conclusions

Though studying the influence of pavement thickness; gradation type; asphalt binder type; and KS content on durability, noise reduction and skid resistance of UTL, the following conclusions can be drawn:

(1) The self-falling impact tester and the corresponding impact freeze thaw split test method developed in this paper can better evaluate the durability characteristics of asphalt mixture. The evaluation index of IFTDD has a good correlation with durability. The importance of different factors affecting durability is: pavement thickness > asphalt type > KS content > gradation. The dense framework structure UTL has better durability than suspended dense structure AC.
(2) The effect of KS additive on the comprehensive performance of UTL pavement is limited. Combining different performance indexes, when the KS content is 0.5%, the pavement performance and economic benefit of the dense framework structure rubber-modified asphalt mixture (UTL AR) are the best.
(3) The importance of different factors affecting noise is: gradation > KS content > asphalt type, whereas the influence of pavement thickness is not significant. When NMAS is the same, the noise reduction of asphalt mixtures between different gradations has an obvious difference. Due to the rich surface texture and pores, UTL pavement had lower noise than that of AC. Additionally, pavement thickness has no obvious effect on noise. When specimen thickness increased from 2 cm to 4 cm, noise only reduced by 0.3 dB.
(4) Due to the high damping coefficient and remarkable effect on absorbing tire/road vibration noise, the rubber-modified asphalt AR mixture has better noise reduction than that of the virgin asphalt and SBS modified asphalt mixture. However, the addition of KS additive can reduce the damping of asphalt mixture and increase the vibration noise of tire-pavement by improving the stiffness of asphalt mixture.
(5) Under the reciprocating action of wheel loading, the skid resistance indexes of asphalt mixture showed rapid attenuation in the initial stage and stable change in the later stage. ‘S-type’ prediction model can effectively simulate the attenuation law of skid resistance. In addition, the skid resistance durability of UTL is evaluated by the long-term benefit index E*_eff_* of pavement safety performance. As a result, the dense framework structure asphalt rubber mixture (UTL AR) has excellent skid resistance durability, which is an extremely important engineering value.
(6) With comprehensive consideration of durability, noise reduction and skid resistance, rubber-modified asphalt UTL pavement with 13.2 mm NMAS (UTL13 AR), thickness of 3 cm and additive content of 0.5% is recommended to applied in field project.

## Figures and Tables

**Table 1:** Main technical indexes of coarse aggregate.

Technical Indexes	Aggregate Size/mm			
	13.2–16	9.5–13.2	4.75–9.5	2.36–4.75
Apparent relative density	2.967	2.964	2.938	2.843
Bulk volume relative density	2.927	2.879	2.877	2.791

**Table 2:** Main technical indexes of three asphalt binders.

Technical Indexes	Penetration/(0.1 mm)	Softening Point/°C	Ductility/cm		Rotational Viscosity/(Pa·s)	
			At 10 °C	At 5 °C	At 135 °C	At 180 °C
Virgin Asphalt	90	46	>100	-	-	-
Asphalt Rubber	62	59.8	-	9.3	3.28	1.03
SBS Modified Asphalt	56	88.7	-	38	1.7	0.275

**Table 3:** The parameter combinations of rut board specimens.

Influence Factors	Parameters of Rut Board Specimen			
	Asphalt	Thickness/cm	Gradation	KS content/%
Gradation Type	Asphalt Rubber	3	UTL10, UTL13, AC10, AC13	0
Asphalt Type	SK-90, SBS, Asphalt Rubber	3	UTL13	0
Content of Admixture	Asphalt Rubber	3	UTL13	0.3, 0.4, 0.5, 0.6
Pavement Thickness	Asphalt Rubber	2	UTL10, UTL13, AC10, AC13	0
		3		
		4		

**Table 4:** Noise test results of asphalt mixture with different gradations.

Gradation	Sound Intensity Level/dB	Sound Pressure Level/dB
AC10	76.2	78.9
AC13	80.4	83.3
UTL10	74.9	77.6
UTL13	77.3	80.1

**Table 5:** Noise test results of UTL with different kinds of asphalt binder.

Asphalt Mixture	Asphalt Binder	Sound Intensity Level/dB	Sound Pressure Level/dB
UTL-13 3 cm	AR	77.3	80.1
	SBS	79.6	82.2
	SK-90	77.9	81.7

**Table 6:** Noise test results of UTL with different KS contents.

Asphalt Mixture	KS Content	Sound Intensity Level/dB	Sound Pressure Level/dB
UTL-13 AR 3 CM	0%	77.3	80.1
	0.3%	77.2	80.0
	0.4%	78.2	81.0
	0.5%	78.7	81.6
	0.6%	80.2	83.1

**Table 7:** Noise test results of asphalt mixture with different mixture thicknesses.

Asphalt Mixture	Thickness/cm	Sound Intensity Level/dB	Sound Pressure Level/dB
AC10 AR	2 cm	76.4	79.1
	3 cm	76.2	78.9
	4 cm	76.7	79.5
AC13 AR	2 cm	80.1	83.0
	3 cm	80.4	83.3
	4 cm	80.3	83.0
UTL10 AR	2 cm	74.4	77.3
	3 cm	74.9	77.6
	4 cm	74.3	77.0
UTL13 AR	2 cm	77.4	80.2
	3 cm	77.3	80.1
	4 cm	77.6	80.4

**Table 8:** The BPN model parameters of different asphalt mixtures.

Function Model	Asphalt Mixture	β	k	R^2^	T/(10^3^ Times)
$BPN=a+\frac{a-b}{1+e^{\beta +\mathrm{kt}}}$	AC10 AR	−0.135	−0.063	0.819	40.98
	AC13 AR	0.173	−0.044	0.807	65.34
	UTL13 SBS	0.248	−0.044	0.810	78.49
	UTL13 SK−90	0.088	−0.057	0.789	61.49
	UTL10 AR	0.347	−0.047	0.739	65.13
	UTL13 AR	0.694	−0.047	0.796	72.54
	UTL13 AR 0.3% KS	0.726	−0.045	0.761	80.09
	UTL13 AR 0.4% KS	0.973	−0.045	0.789	76.99
	UTL13 AR 0.5% KS	0.984	−0.046	0.807	81.99
	UTL13 AR 0.6% KS	0.841	−0.041	0.819	87.42

**Table 9:** The TD model parameters of different asphalt mixtures.

Function Model	Asphalt Mixture	β	k	R^2^	T/(10^3^ Times)
$TD=a+\frac{a-b}{1+e^{\beta +\mathrm{kt}}}$	AC10 AR	−2.056	−0.146	0.829	13.88
	AC13 AR	−0.590	−0.100	0.807	35.02
	UTL13 SBS	−0.570	−0.088	0.825	46.99
	UTL13 SK−90	−0.938	−0.164	0.816	39.18
	UTL10 AR	0.736	−0.129	0.843	37.40
	UTL13 AR	1.192	−0.123	0.822	42.94
	UTL13 AR 0.3% KS	1.412	−0.111	0.777	49.50
	UTL13 AR 0.4% KS	0.852	−0.112	0.814	48.05
	UTL13 AR 0.5% KS	0.867	−0.115	0.891	47.09
	UTL13 AR 0.6% KS	0.832	−0.110	0.765	48.91

**Table 10:** Long term benefit analysis of skid resistance indexes of asphalt mixture.

Mixture Types	BPN				TD			
	T_BPN_/10^3^	*E_avg_*	*E_eff_*/%	Rank	T_TD_/10^3^	*E_avg_*	*E_eff_*/%	Rank
AC-10 AR	40.98	45.23	22.3	10	13.88	0.64	16.4	10
AC-13 AR	65.34	49.22	33.0	9	35.02	0.69	25.5	8
UTL-13 SBS	78.49	51.63	39.5	4	46.99	0.74	34.5	5
UTL-13 SK-90	61.49	49.61	34.1	7	39.18	0.69	25.5	8
UTL-10 AR	65.13	49.39	33.5	8	37.40	0.70	27.3	7
UTL-13 AR	72.54	50.62	36.8	6	42.94	0.73	32.7	6
UTL-13 AR 0.3% KS	80.09	51.38	38.9	5	49.50	0.77	40.0	1
UTL-13 AR 0.4% KS	76.99	52.54	42.0	1	48.05	0.77	40.0	1
UTL-13 AR 0.5% KS	81.99	52.32	41.4	2	47.09	0.76	38.8	3
UTL-13 AR 0.6% KS	87.42	51.87	40.2	3	48.91	0.75	36.4	4

**Table 11:** Results of ANOVA of different factors on durability, noise and skid resistance.

Influencing Factors	Dependent Variables							
	Durability		Noise		BPN		TD	
	F	P	F	P	F	P	F	P
Pavement Thickness	1851.942	<0.001	2.793	0.114	-	-	-	-
Asphalt type	1744.118	<0.001	34.935	<0.001	35.317	<0.001	2.387	0.147
Gradation	41.550	<0.001	133.59	<0.001	34.648	<0.001	41.867	<0.001
KS content	266.952	<0.001	48.390	<0.001	101.194	<0.001	8.371	0.001
